# Comparing the effect of humanoid and human face for the spatial orientation of attention

**DOI:** 10.3389/fnbot.2013.00012

**Published:** 2013-09-03

**Authors:** Thierry Chaminade, Maria M. Okka

**Affiliations:** Institut de Neurosciences de la Timone, Aix-Marseille Université - Centre National de la Recherche Scientifique UMR7289Marseille, France

**Keywords:** social cognition, attention, humanoid robot, posner cuing task, reaction time

## Abstract

The current study was designed to investigate how the automatic spatial orientation of attention induced by the perception of another agent's orientation of attention is modulated by the social nature of the other agent. Modified versions of the Posner task, using a real or schematic face with eyes or head looking toward the left or the right before a to-be-detected target appears on one side of the screen have been used to demonstrate a reduction of reaction time (RT) for target detection when the gaze is directed toward the target, even though the cue is not informative. We compared the effect of two agents, the humanoid robotic platform Nao and a real human, using head turn to cue the spatial orientation of attention. Our results reproduced the typical Posner effect, with reduced RT to valid compared to invalid spatial cues. RT increased when no spatial information was provided, interpreted as an increased difficulty to disengage from a direct gaze. RT was also increased when the robot was used instead of the human face and when the eyes of the stimuli were blacked out. Both effects were interpreted as resulting from an increased difficulty to disengage attention from the central stimulus because of its novelty. In all experiments, there was no interaction between cue validity and cue agent, implying that the exact nature of the human-like agent didn't have an effect on the automatic spatial orientation of attention. Altogether, our results imply that a humanoid face is as potent as a human face to trigger an automatic orientation of spatial attention.

## Introduction

Autism is a neurodevelopmental disorder characterized by impairments in communication, social interactions and behaviors. According to the social motivation hypothesis (Chevallier et al., 2012), deficits in social cognition would be the consequence of an extreme disruption of social interest, encompassing a lack of preference for social stimuli and an absence of reward signal during social interactions. Current care strategies attempt to teach adapted skills soon after diagnosis, and intensive early intervention can significantly improve the communicative skills in pre-school children. But these interventions rely on the use of human therapists, and the use of robots in alternative therapies of autism has been under scrutiny for decades (Robins et al., 2004). In particular, the developments in the field of humanoid robotics, corresponding to robots that have human-like features such as face, hands or other body parts, was largely supported by the proposal that they could replace human therapists in early intervention programs. The underlying assumption was that these agents would both bypass autistic children's avoidance of social interaction and make use of their attraction for predictable objects. Meanwhile, these robots with human-like appearance would possess all necessary features to convey social signals: a face with mouth to express emotions, eyes to inform about direction of attention, arms, and hands to support physical interactions. Anecdotal observations notwithstanding (Kozima et al., 2004, 2007), there is a lack of empirical studies in support of the idea that these robots would be beneficial in autism therapies (Diehl et al., 2012), and directly addressing this assertion with tools of social cognitive neuroscience is at the core of our research program (Chaminade and Cheng, 2009).

Joint attention, when two (or more) individuals pay attention to the same object or location in space, is a key behavior for social interactions that is impaired in autism (Charman, 2003). It was suggested, but on the basis of unstructured observations, that Keepon, a creature-like robot capable of expressing its attention (gaze direction) and emotion (using motion) facilitates the emergence of joint attention in patients-caregivers dyads (Kozima et al., 2007). It was even reported one observation that an autistic child “drew the gaze-line” to figure out the direction of the humanoid robot “infanoid” attention (Kozima et al., 2004), an example of how robots could be used to teach to “read” explicitly the direction of attention with a robot. Interestingly, neuroscience also supports the idea that humanoid robots could be used to rehabilitate social abilities in children with autism, as the same brain region, the posterior superior temporal cortex, is involved in processing social cues from the observation of others' gaze (Allison et al., 2000), has been repeatedly shown to have abnormal activity in tasks involving social cognition in autism (Saitovitch et al., 2012), and is sensitive to the perceived humanness of an artificial agent (Chaminade et al., 2007).

One fundamental component of joint attention is the automatic orientation of attention elicited by the perception of another individual attending to an object or a location in space. This automatic orientation of attention includes an “Eye Direction Detection” (EDD) mechanism (Baron-Cohen, 1997) for detecting the presence of eye and inferring the spatial direction of attention of the basis of simple visual cues. This EDD, present in neonates (Batki et al., 2000), would be necessary for the development of more complex social processes such as joint attention and intention detection (Baron-Cohen, 1997). An experimental psychology paradigm testing this automatic orientation of attention is a modified version of Posner's spatial orienting task (Posner, 1980) using social cues. In the classical Posner's task, participant must report the apparition of a target on the left or right of a central fixation. Before target presentation, a spatial cue, an arrow pointing to the left or right, can be provided. If the target appears in the cued location (i.e., left target when the arrow pointed to the left of the central fixation), responses are typically faster, indicating that the participant's attention has been shifted to this spatial location (the left side of the screen). If the target appears on the opposite side, then the person must shift attention from the cued to the opposite side, slowing down the response. The measured effect is the variation in reaction time (RT) to targets appearing in the cued location (valid cues) and the non-cued location (invalid cues). The facilitation of target detection in valid cue condition is largest for intervals between cue and target apparition of *circa* 300 ms (Cheal and Lyon, 1991). When the cue is not informative, meaning it is as likely to be valid than to be invalid, its effect on RT is caused by an automatic orientation of attention toward a spatial location.

The Posner task has been adapted to study the EDD. Instead of an arrow, a direction in space is primed by a social cue indicating where this person is attending. Participants are faster to respond when the social cue indicates the location on the screen where the target appears even when this social cue is not informative. Experiments used a real (Driver et al., 1999) or a schematic (Friesen and Kingstone, 1998) face with eyes looking toward the left or the right of the screen. In both reports, a clear advantage of congruent spatial cues for target detection was found, also with a SOA of 300 ms. A variant of the Simon task using schematic drawings of eyes (Zorzi et al., 2003) confirmed the automaticity of the processing of gaze direction. Participants responded with left or right buttons to the color of the “iris,” that appeared to be looking straight, left or right. The classical Simon effect was reported, a reduced RT when the direction of the schematic gaze is congruent with the side of the button that has to be pressed even though it is not informative. Furthermore, the use of faces with negative black and white polarities demonstrated that the automatic orienting of attention by perceived gaze is triggered by the unique human eye morphology, with a white sclera surrounding a darker iris (Yoshizaki and Kato, 2011). The body posture can also be processed automatically as a cue for the spatial orienting of attention. An experiment used a Posner task varying the orientation of the torso and of the head, always with directed gaze. There was an effect of the orientation of the head toward a direction in space while the body was in front view but not when it was also oriented to the side (Hietanen, 2002), indicating that it was the spatial direction of attention relative to the body frame of reference of the other person (i.e., the other person is not *turned* to the left, it is *looking* toward *her* left) that triggered the facilitation effect, strongly suggesting that the information that is being automatically processed is social (“direction of attention”) and not purely spatial (“orientation of the body”).

Interestingly, it is not clear whether this automatic orienting of attention by social cues is impaired in autism. In one report using a schematic face with high-functioning autism patients, only the controls showed the facilitation effect of congruent cues at 300 ms (Ristic et al., 2005). But another study found no such difference using a real face gazing to the left and the right in children with autism matched with controls in mental-age (Kylliainen and Hietanen, 2004), and a review of the literature indicates that the majority of studies failed to replicate an impairment for this task in autism (see Table 1 in Nation and Penny, 2008). On the other hand, the finding that controls, but not autistic patients, are faster to disengage from geometrical than social cue has been reported in several experiments (Chawarska et al., 2003; Senju et al., 2004; Vlamings et al., 2005). Comparing response time between a humanoid robot and a real face in such a task could help dissociate between the avoidance of real human vs. an effect of human shape in this facilitated disengagement.

It is known that humans can interpret cues provided by humanoid robots to convey information about their object of attention (Kozima et al., 2007; Staudte and Crocker, 2009; Boucher et al., 2012). One study investigated the automatic orientation of attention by a humanoid robot, but it used among other cues both a very human-like robot Zeno and the minimalist robot Keepon (Admoni et al., 2011), as well as a complex variation of the spatial orientation task varying the predictability of the cue. The present study investigated how early processes of social attention orienting are preserved when a robotic humanoid face turning from the upper torso is used instead of a real human in neurotypical adults.

## Materials and methods

The main paradigm was adapted from classical experiments (Driver et al., 1999; Senju et al., 2004). The stimuli were inspired by those used by Hietanen (2002) to investigate body posture cues. All experiments adhered to the Declaration of Helsinki. Participants signed informed consent.

The experiments were run on a Dell PC laptop (Windows XP) with a 19-inch LCD color monitor using Paradigm-Experiments for stimuli presentation and data collection. The participant's RT was measured with a custom-made response button held in his dominant hand. The experiments took place in a dark room where the participant was seated ~60 cm from the screen.

A central cross measuring 1.2 cm on the monitor was used as a fixation point. Human stimuli were color pictures of the head and the upper torso of a male model in three different positions: front view oriented straight ahead, a front view of the torso with the head oriented 40° to the left and with the head oriented 40° to the right. Robot stimuli consisted of color pictures of the upper torso humanoid robot Nao T14, in the same different positions as the human stimuli. The front views were used both as precues and cues. The human and the robot stimuli both had a straight gaze, a neutral expression and measured, respectively, 10.8 cm at the widest and 10.1 cm high (~10° visual angle in both dimensions). The target stimulus consisted in the central cross spanned by an asterisk measuring 0.5 cm and positioned at 5.2 cm from the central fixation cross on either the left or the right side of the image.

**Figure 1:**
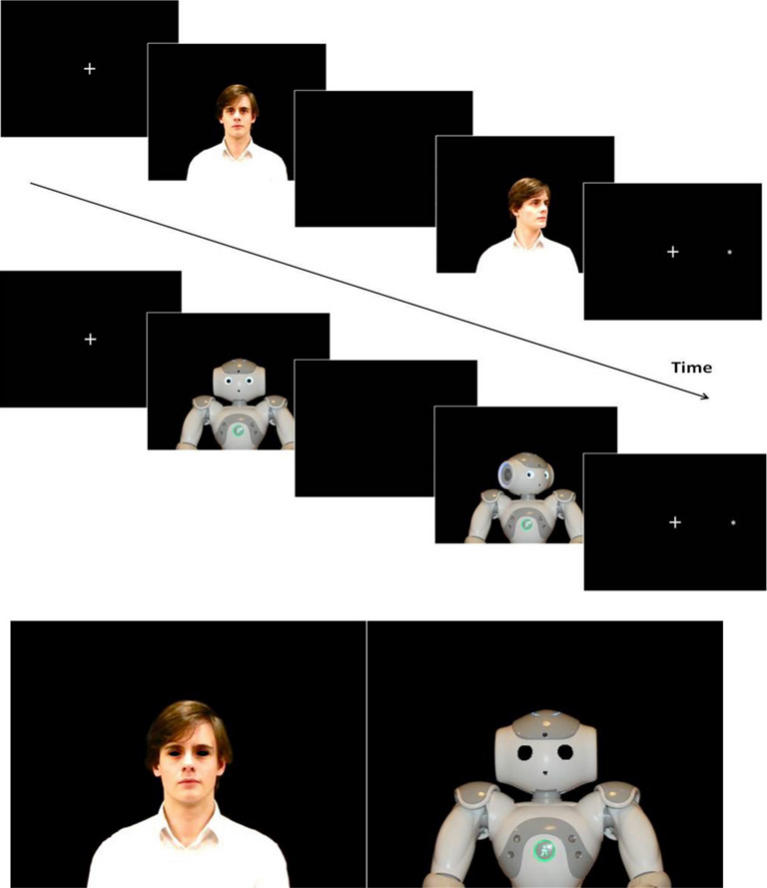
**Top:** Schematic representation of the sequence of events in a trial defined by the target appearing on the right of the agent (**top**: human, **bottom**: robot) and a valid cue. **Bottom:** Neutral stimuli used in experiment 2.

Each trial contained the same sequence of events (illustrated in Figure 1) that consisted of the following: fixation cross for 675 ms; precue stimulus (human or robot face front view) for 900 ms; black screen for 30 ms; cue stimulus (face front view or face oriented 40° to the left or right with torso front view) for 300 ms; target stimulus (or fixation cross for catch trials), that remained visible until response for a maximum of 1 s. Participants were instructed to fixate the center of the monitor and to maintain their fixation throughout the experiment. They were asked to press the response button as soon as they detected the appearance of the target. It was specified to participants that the agent's head orientation was irrelevant to the target position. In catch trials, the same sequences of precue and cue stimuli than for experimental conditions were used, leading to 6 types of catch trials.

### Experiment 1

Fourteen right-handed individuals (4 men; mean age, 27 years; range, 22–49 years) naïve as to the purpose of the experiment with normal or corrected-to-normal vision volunteered to participate in this experiment. There were 12 experimental conditions defined by three within-subjects factors: the *cue agent* (human or robot), the *cue validity* and *target laterality*. The second factor corresponded to the spatial location of the cue and target stimuli: the cue was valid when the target appeared on the cued side (left cue, left target and right cue, right target), invalid when it appeared on the uncued side (left cue, right target and right cue, left target), and neutral when the cue showed the robot or human front view, therefore providing no spatial information.

A session was composed of 90 trials in random order, with 72 test trials and 18 catch trials (6 repetitions of all types of experimental trials). The four sessions were separated by short resting times. Prior to the experiment, a practice session including 18 trials (one example of each of the 12 experimental conditions and of each of the 6 types of catch trials) was presented. Each session lasted ~4 min, so that the overall duration of the experiment, including the practice session, was approximately half an hour.

### Experiment 2

Fourteen right-handed participants with normal or corrected-to-normal vision, including 4 that took part in Experiment 1 (7 men; mean age 23.9 years; range 20–36 years; right-handed) participated in this experiment. The experimental paradigm was the same as in Experiment 1 except that the eyes of each both agents were blacked out (Figure 1). The same color as the background (black) was used to mask the agents' eyes. Picture editing focused on removing the white sclera and the iris, keeping the rest of the eye region intact (Driver et al., 1999). The same different positions as those adopted by the human and the robot in the Experiment 1 (front view oriented straight ahead, a front view of the torso with the head oriented 40° to the left and with the head oriented 40° to the right) were used in this second experiment. (Illustrated in at the bottom of Figure 1). All other features of experiment 1 were kept.

### Experiment 3

Experiment 3 manipulated the congruence between the agent presented in the precue and cue stimuli. Twenty-four participants with normal or corrected-to-normal vision who didn't take part in the two previous experiments (6 men; mean age, 23.3 years; range, 19–44 years; 19 right-handed and 5 left-handed) were tested in this experiment. We used the same stimuli as in Experiment 1. In Experiments 1 and 2, the precue stimuli were always followed by a cue that presented the same agent, while in the third experiment, the precue and cue stimuli were incongruent in half of the trials. As there were 24 experimental conditions defined by four within-subjects factors: the precue agent (human or robot), the cue agent (human or robot), the cue validity (neutral, invalid, and valid) and target laterality (left or right). To compensate for the increase in the number of experimental conditions, a session was composed of 96 trials, with 72 test trials and 24 catch trials and six test sessions were recorded.

### Analysis

RTs corresponding to low and high outliers were removed from the data analysis with a technique of outliers detection and treatment using a modified square-root transform (Cousineau and Chartier, 2010). For each participant, the modified square-root transformation was applied in order to locate outliers at either side of the decile distribution of transformed data, deleting extreme values iteratively (i.e., at either end of the distribution, separated of the central distribution by an empty decile) iteratively. Thus, each dataset corresponded to a normal distribution.

The data were analyzed with a five-way mixed-effects analysis of variance (Statistica™). Because they used exactly the same paradigm and to be able to compare RTs, experiments 1 and 2 were analyzed together. Subjects, sessions and trials were treated as random factors and cue agent (2: human or robot), and cue validity (3: neutral, invalid, valid) as well as experimental paradigm (2 levels: eyes visible and eyes hidden) for experiments 1 and 2 and precue agent (2: human or robot) for experiment 3, as factors of interest. Target laterality was also included as factor of interest to assess whether the apparition of the target on the left or right influenced RTs, but no interaction with other factors was predicted and therefore computed. Thus, in addition to main effects, only interactions between the other variables of interest were calculated.

## Results

### Experiments 1 and 2

**Figure 2:**
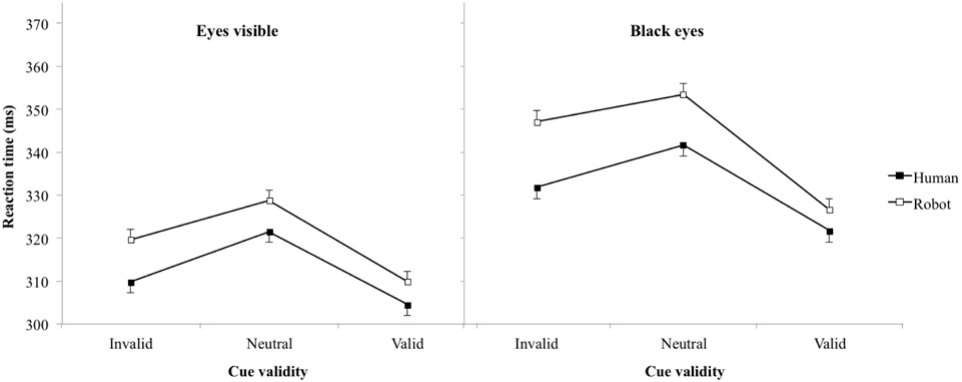
**Reaction time (error bar: standard errors of the means) plotted against cue validity for human cue test and robot cue test in experiments 1 (eyes visible) and 2 (blacked-out eyes)**.

On average, participants produced false alarms in 9.6% of catch trials. Table 1 and Figure 2 present mean RT (and standard error of the mean). Laterality of the target excepted [*F*_(1, 8012)_ = 3.0, *p* = 0.08; η^2^ = 0.01], main effects for variables of interest were all significant: cue agent [*F*_(1, 8012)_ = 27.1, *p* < 0.001, η^2^ = 0.09], cue validity [*F*_(2, 8012)_ = 48.1, *p* < 0.001, η^2^ = 0.31], and paradigm [*F*_(1, 8012)_ = 155.6, *p* < 0.001, η^2^ = 0.51], while none of the interactions reached significance (all *p*s > 0.15). Excluding from the analysis the four participants that were tested in both paradigms didn't affect qualitatively the significativity of the effects. Response time for the robot was significantly longer than for the human (330 vs. 322 ms) and for the eye hidden than the eye visible paradigms (337 vs. 316 ms). *t*-tests showed that pairwise comparisons between all pairs of cue validity were significant (*p* < 0.001), with the longest RT for neutral and the shortest RT for invalid cues (valid 316 ms, neutral 336 ms, invalid 327 ms).

**Table 1:** **Reaction time in ms (standard errors of means) as a function of the cue agent and eye visibility**.

	**Paradigm**	**Eyes visible**		**Black eyes**	
	**Cue agent**	**Human**	**Robot**	**Human**	**Robot**
Cue validity	Neutral	321 (2)	329 (2)	342 (2)	353 (2)
	Invalid	310 (2)	320 (2)	332 (2)	347 (2)
	Valid	304 (2)	310 (2)	322 (2)	326 (2)

### Experiments 3

**Figure 3:**
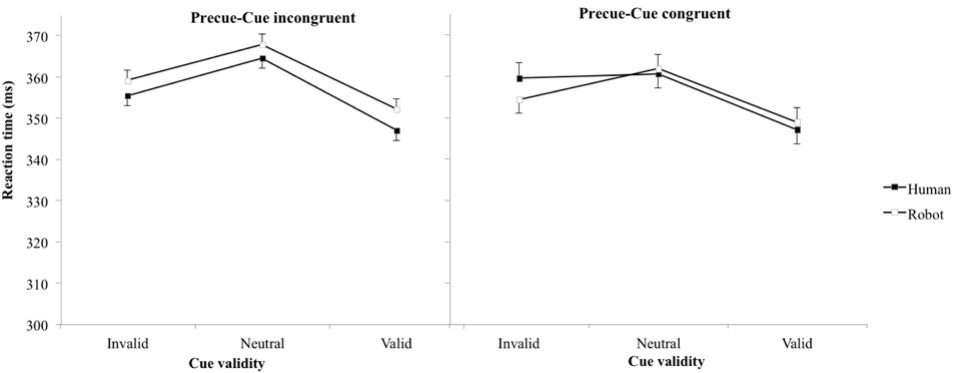
**Reaction time (error bar: standard errors of the means) plotted against cue validity for human and robot cues when pre-cue and cue stimuli are congruent (left) and incongruent (right)**.

On average, participants produced false alarms in 7.6% of catch trials, with no significant differences between experimental conditions. Table 2 and Figure 3 present mean RT (and standard error of the mean). The main effect of cue validity was reproduced [*F*_(2, 6283)_ = 26.7, *p* < 0.001, η^2^ = 0.57], but no other main effects nor interactions reached significance (all *p*s > 0.15). *t*-tests showed that pairwise comparisons between all pairs of cue validity were significant (neutral-invalid *p* = 0.02, neutral-valid *p* < 0.001, valid-invalid *p* = 0.002), with the longest RT for neutral and the shortest RT for valid cues (valid 349 ms, neutral 364 ms, invalid 357 ms).

**Table 2:** **Mean reaction time in msec (standard errors of the mean) as a function of cue and precue agents**.

	**Cue agent**	**Human**		**Robot**	
	**Precue agent**	**Human**	**Robot**	**Robot**	**Human**
Cue validity	Neutral	364 (3)	361 (4)	368 (4)	362 (3)
	Invalid	355 (4)	359 (3)	359 (4)	354 (3)
	Valid	347 (3)	347 (3)	352 (3)	349 (3)

## Discussion

Exogenous orientation of attention by social stimuli representing upper torso with heads turned to attend specific spatial locations of the environment (Driver et al., 1999; Hietanen, 2002; Senju et al., 2004) was used to investigate whether a humanoid robotic platform would differentially affect this automatic processing compared to a real human face.

### Effect of cue validity

A significant main effect of cue validity was found in all experiments. In particular, the RT in invalid conditions (when the target appeared in the opposite of the cued side) was higher than the RT in valid conditions (when the target appeared in the cued side). We therefore reproduced the typical response facilitation on trials with valid relative to invalid spatial cues. This finding confirmed that a central and unpredictive cue, in our case a human or robot face turned to the side, with (Experiment 1) or without (Experiment 2) the eyes being visible could trigger a shift of the observer's spatial attention.

RTs were systematically longer for neutral conditions, corresponding to the agent keeping its face in front view at the cue onset, than when the agent turned its head, when this spatial cue is valid but also when it is invalid. In a pilot experiment the mean RT for the neutral cue seemed abnormally high (430 ms). We speculated that the apparition of the head turned to the left or right served as a temporal information for the onset of the target, while the same image was used for precue and cue in the neutral condition; this information could be used to prepare the peripheral allocation of attention with a better temporal accuracy and consequently reducing RT in both valid and invalid trials. A 30 ms black screen was therefore added between precue and cue images to provide the same temporal information (perceived as a flash) in all conditions. The increase of RT for the neutral conditions was still present, implying that the differences in RT between neutral and either valid or invalid spatial cues were not an artifact of the experimental paradigm. This effect has been reported in previous experiments, and was interpreted as reflecting the absence of a congruent spatial cues (Hietanen, 2002). The fact that response to neutral trials was significantly longer than in invalid trials, in which the cue provides information that should interfere with spatial allocation of attention and therefore increase RTs, suggested an alternative interpretation.

As this version of the Posner task investigated automatic processes involved in social interaction, we propose a social interpretation of this effect, namely that it is harder to detach one's attention from the center of the screen when it presents a face looking directly at you (Senju and Hasegawa, 2005). This effect seems to integrate information given by the eyes, interpreting the respective positions of the iris, but also the body posture, provided that is survived when the eyes were not visible. In agreement with this interpretation, we argue that increases in RTs independent of cue validity in this experiment reflect a reduction in the ability to shift attention from central cues to detect peripheral targets. In other words, the effects of cue validity on RT reflect spatial orientation of attention, while the other effects affecting RT are interpreted in terms of disengagement from the central cues.

### Effect of eye visibility

Social cues like body posture including head orientation (Hietanen, 1999) and gaze direction (Jellema et al., 2000) are integrated in a hierarchical way (Oram and Perrett, 1996). The direction of the eyes provides a better indication on the others' direction of attention than the head orientation, itself a better cue than body posture. But Langton (2000) suggested that when all these cues are visible, specifically when head direction and gaze direction can be seen, the information about spatial direction of attention direction is automatically extracted from both of them, each cue giving an independent signal. In the present case removing the eyes information slowed down target detection altogether (left and right graphs in Figure 2 have the same ordinate scale). The spatial orienting of attention wasn't faster when the eyes were present than when they were absent, in the case of human stimuli (this would have been reflected in a significant three-way “paradigm” by “cue agent” by “cue validity” interaction). We also expected a reduction of the effect seen in the neutral condition provided the eyes are particularly important in catching attention (Langton et al., 2000), but as discussed in the previous section this was not observed.

An alternative explanation of the difficulty to disengage from the stimuli with blacked-out eyes could be that they are “uncanny.” Removing the eyes from a face is particularly disturbing, in that it breaches the expectations from our perceptual system. In a previous fMRI experiment investigating the “Uncanny Valley” phenomenon, i.e., the fact that android robots are repulsive, we interpreted this breach of expectation as the source of signals that catch attention (Saygin et al., 2012). While the uncanny effect could have been stronger for the human that robotic head, as we have visual expertise for the former but not the latter, no interaction with the agent was found.

### Effect of agent

Irrespective of cue validity, targets appearing following human cues were detected significantly faster than targets appearing following robot cue. It is the first time that a significant slowing down in a RT task is reported for a robot compared to human stimuli in a Posner spatial orientation task (no effect of stimulus in Admoni et al., 2011). Some participants in Experiment 1 reported that they felt that the robot, but not the human, was staring at them whatever its posture. This illusion was due to the larger area of brighter color surrounding the darker center of the robot compared to the human eyes could be detected automatically (see Box 2 in Langton, 2000), so what was perceived as the equivalent of the iris could be seen as turned toward the center of the image instead of to the side, and therefore looking at the participant. It is in full agreement with the finding that humans' prior expectation that gaze is directed toward them (Mareschal et al., 2013). Following our interpretation of the neutral cue effect, this could have explained an increased difficulty to disengage from robot than from human stimuli leading to increased RTs. We tested this possibility with a second experiment in which the inside of the eyes were blacked out to remove eye gaze information about the object fixated by the cue that could have been processed by the relative positions of the iris and sclera. Four of the participants that reported the illusion with the unedited stimuli were retested. We found a main effect of the experimental paradigm when comparing the stimuli with normal or blacked-out eyes, but the main effect of the agent survived, and in both experiments RT to the robot was longer than the human stimuli, with no significant interaction between the presence of the eyes and other experimental factors (cue agent, cue validity) or interactions between them (cue agent by cue validity).

As for the neutral stimuli, the increased RT for robot stimuli signaled a difficulty to disengage from the centrally presented stimulus. The interpretation of this finding is that it is more difficult to disengage one's attention from a humanoid robot than from a human, whatever the posture of the agent. Several interpretations of this data can be presented at this stage, and further work is required to disentangle them. In particular, one can speculate why human and robot cues differed in their ability to attract attention, in particular the fact that they could have significantly different low-level visual features that can influence the allocation of attention, but a lot of importance was given to the preparation of the stimuli. First, great care was taken to equate the field of view between the agents. Second, the position of the eyes and necks were carefully equated when preparing the stimuli, despite the differences between the overall shapes of the two heads. Finally, the human was by nature brighter and more contrasted than the robot and should have attracted attention more. Altogether, we propose that the highly significant and reproducible increase of RT is better explained by the nature of the agent than by the low-level visual characteristics of the stimuli.

The social nature of a humanoid robot is inherently ambiguous: it is clearly a machine, yet it reproduces human facial features important for communication, and in particular the eyes. Interestingly, an increase of RT was found when comparing human to geometrical spatial cues in children and adults (Chawarska et al., 2003; Senju et al., 2004; Vlamings et al., 2005), which was interpreted as an increased difficulty to disengage from a real human face compared to geometrical cues because it is a social signal. Were the robot treated by the perceptual system purely as a geometrical cue, we would expect to reproduce this effect and find a reduced RT for the robot compared to the human. Results indicate the opposite. We can interpret this by considering that the spatial cue is interpreted not as geometric, but as social. It should first be noted that there is no simple geometrical cue oriented toward a direction in space in the stimuli, therefore that can only be interpreted in anthropomorphic terms (“*turning the head*”). More importantly, we previously suggested that robotic devices might automatically attract attention because they do not match perfectly our perceptual filters for the perception of human agents (Saygin et al., 2012). In this framework, our social perceptual system is shaped by our experience in life, in which we mostly perceive real human faces. The early recognition of a human face (early both in development and neural processing) facilitates further processing of facial features (identity, emotion or, in the current experiment, orientation of attention). But as a robotic face only imperfectly reproduces the human face, the early stage of facial recognition triggers error signals (“this resembles a face but doesn't match face prototype”) that slow down further processing of the stimulus. In other words, humanoid robots are social because of their their human-like shapes, but the fact that they are not perfectly humans hinders this automatic extraction of mental states their appearance which recruits additional attentional resources to process (Chaminade et al., 2010; Saygin et al., 2012). Accordingly in the present experiment, all conditions are similarly slowed down because all required a similar disengagement from the central robot stimulus.

### Effect of congruency between precue and cue agents

We designed the third experiment to test whether we could separate the effect of this hindered disengagement of attention from the center of the screen with robot compared to human cues from the spatial orientation of attention. We hypothesized that the long duration of the neutral robot precue would trigger an increased RT irrespective of the agent used for the spatial cue. Statistically, we expected an effect of the agent used as pre-cue, but not of the agent used as cue, in the RT. Results failed to confirm this hypothesis. The congruency effect was reproduced indicating that the orientation of attention by agents' orientation was preserved, but no other experimental factor significantly influenced RTs. Some remarks can be made from the observation of Figure 3. First, RTs were higher than for experiments 1 and 2 (the same ordinate scales are used in the four graphs for direct visual comparison). Then, when focusing on congruent conditions (left panel) that corresponded to the exact same conditions than experiment 1, the RT for robot were still longer than for human (compare with left panel, Figure 2). Finally, the one point that stood out from other results (condition Incongruent, Human, Invalid), with longer RT than would be expected, was when a robot precue precedes a human cue, as hypothesized. Nevertheless, the absence of statistical significance suggested that the precue and cue stimuli were not processed independently enough to disentangle their respective effects with this paradigm. On the other hand, the incongruence between the agent used as precue and cue stimuli seemed sufficient to affect attention disengagement (higher RTs). Altogether, response times when the agent used as precue and cue were not congruent corresponded to complex interactions between, and not simple addition of, cognitive processes.

## Conclusion

This experiment investigated whether the intentional nature of an agent, a human or a humanoid robot (in the present case, Nao), influenced the exogenous orientation of attention by spatial cues indicating the direction of attention using orientation of the head relative to the torso. Despite a main effect corresponding to a significant and reproducible increase of RT when the spatial cue was provided by the robot, no interaction between spatial orientation and agent were found. This result implies that a humanoid robot triggers the same mechanism of automatic orientation of attention than a human. More interestingly, we had a similar interpretation to explain the increase of RT for the robot compared to the human cue, as well as for all other significant effects: RT to neutral stimuli was significantly longer than to both valid and invalid conditions, RT to stimuli with eyes blacked out was also longer than to the unedited stimuli, RT in the experiment including incongruences between precue and cue stimuli were longer than in experiments without incongruence. In all cases, we proposed that this increase reflected the fact that the central stimulus grabbed attention, because the eyes were directed toward the participants, because the cue was a robot a robot, because the blacked-out eyes were uncanny, because the precue was not predictive of the cue. This would be in line with previous conclusions that cognitive responses to humanoid robots involve an increase of attention that is triggered by their novelty in contrast to their overall human shape.

### Conflict of interest statement

The authors declare that the research was conducted in the absence of any commercial or financial relationships that could be construed as a potential conflict of interest.
